# A prospective case–control pilot study to evaluate bone microarchitecture in children and teenagers on long-term parenteral nutrition using HR-pQCT

**DOI:** 10.1038/s41598-021-88366-6

**Published:** 2021-04-28

**Authors:** Typhaine Louazon, Pierre Poinsot, Lioara Restier, Abdelouahed Belmalih, Irène Loras-Duclaux, Stéphanie Marotte, Sophie Heissat, Didier Barnoud, Cécile Chambrier, Cyrille B. Confavreux, Alain Lachaux, Justine Bacchetta, Noel Peretti

**Affiliations:** 1 Hospices Civils de Lyon, Department of Pediatric Hepato-Gastroenterology and Nutrition, Hôpital Femme Mere Enfant HFME, 59 Bd Pinel, 69677 Bron, France; 2 Univ Lyon, UCBL 1, Lyon, France; 3 Hospices Civils de Lyon, Department of Intensive Clinical Nutrition, Centre Hospitalier Lyon Sud, 165 chemin du Grand Revoyet, 69495 Pierre-Bénite, France; 4 Hospices Civils de Lyon, Department of Rhumatology, Centre Hospitalier Lyon Sud, 69495 Pierre Bénite, Lyon, France; 5 INSERM U1033, LYOS, Lyon, France; 6 Hospices Civils de Lyon, Reference Center for Rare Diseases of Calcium and Phosphate, Hôpital Femme Mère Enfant HFME, 69677 Bron, France; 7 INSERM U1060, INRA U1397, INSA Lyon, CarMeN laboratory, Charles Merieux Medical School, Univ-Lyon, 69600 Oullins, France

**Keywords:** Diseases, Gastroenterology, Rheumatology

## Abstract

Long-term parenteral nutrition (PN) may induce bone complications. Tridimensional bone imaging techniques such as high-resolution peripheral quantitative computed tomography (HR-pQCT) allow the assessment of both compartmental volumetric densities and microarchitecture. Our aim was to evaluate these parameters in children and teenagers receiving long-term PN. This cross-sectional, case–control study included children older than 9 years undergoing PN for at least 2 years. They were age-, gender- and puberty-matched with healthy controls (1:2). Evaluation included biological assessment of bone metabolism (serum calcium, phosphate, and albumin; urinary calcium and creatinine; 25-OH vitamin D, osteocalcin and PTH), dual X-ray absorptiometry (DXA) and HR-pQCT at the ultradistal tibia and radius. Results are presented as median [range]. Eleven patients (3 girls) with a median age of 16 [9–19] years were included. Bone parameters assessed by HR-pQCT at the ultradistal radius and tibia were similar in patients and controls. Parathyroid hormone (PTH) levels were higher (14 [7–115] vs 16 [12–27]) and osteocalcin levels were lower (44 [15–65] vs 65 [38–142]) in patients than in controls, although within the normal range. Conclusions: there were no differences for compartmental bone densities and microarchitecture in patients undergoing chronic PN. Further longitudinal studies are required to confirm these quite reassuring preliminary results.

## Introduction

Long-term parenteral nutrition (PN) is the most efficient way to prevent malnutrition in patients with severe intestinal failure (IF). However, PN may induce complications such as bone impairment, also called PN-associated metabolic bone disease (PN-MBD)^1–6^. PN-MBD was first described in 1980^5,7^. It is associated with osteoporosis and impaired mineralization^8^. Very few data are available on its exact prevalence, but it is estimated to range from 40 to 90% in adults and from 25 to 80% in children and teenagers^9–12^.

PN-MBD may be associated with metabolic abnormalities such as hypercalcemia, hypercalciuria, acidosis and hypophosphatemia. Even though the pathophysiology of PN-MBD remains to be fully determined, different parameters are nevertheless known to increase the risk of PN-MBD, namely inadequate PN composition (calcium, phosphate, and lactate), the presence of an underlying inflammatory disease, and treatment with corticosteroids.

Teenagers are particularly at risk for PN-MBD because of growth and acquisition of 25% of bone mass during puberty (and 90% over the first two decades of life). However, there are very limited data on long-term bone impairment in children and teenagers undergoing long-term PN.

Dual X ray Absorptiometry (DXA) is the most used technique to assess bone quantity; it is recommended to assess the longitudinal bone health for children at risk^13^. Total bone mineral content (TBMC) must be used for bone assessment according to these recommendations, expressing *z* scores with reference data based at least on age and gender. However, DXA has some technical limitations, especially in growing children and teenagers^13–15^: (i) DXA measures areal density rather than volumetric density that can be modified by growth; (ii) it is unable to evaluate separately trabecular and cortical bone that are sometimes impaired independently; and (iii) DXA cannot determine bone microarchitecture, which is a major determinant of the risk of bone fracture^16^.

Therefore, innovative tridimensional and non-invasive bone imaging techniques have been developed in the early 2000s, such as high-resolution peripheral quantitative computed tomography (HR-pQCT), allowing the assessment of both compartmental (i.e. total, cortical and trabecular) volumetric densities and trabecular microarchitecture in vivo^14,17–20^*.* This improved bone assessment, especially in children and teenagers whose bones continually grow in mass, size, and shape. To our knowledge, HR-pQCT has never been studied in pediatric patients receiving long-term PN.

We hypothesized that children and teenagers receiving chronic PN may display significant bone microarchitecture differences compared with controls matched for age, gender and pubertal status.

The objectives of this pilot study were (1) to determine the density and bone microarchitecture in children and teenagers receiving long-term PN compared to healthy controls, using HR-pQCT; (2) to compare HR-pQCT results to DXA measurements; and (3) to evaluate the biological markers of bone metabolism.

## Patients and methods

This cross-sectional, comparative case-controlled pilot study was performed in the home parenteral nutrition pediatric unit of a university pediatric hospital between March 2014 and June 2015.

### Patients

Inclusion criteria were children receiving home PN for more than 2 years for intestinal failure, which seems a sufficiently long period to evaluate the impact of PN on bone metabolism; older than 9 years of age due to technical limitations of HR-pQCT (need to have a perfectly still child), and with regular follow-up in a university HPN center. Exclusion criteria were congenital bone diseases (2 patients with McKusick syndrome) and corticosteroid use for more than one month during the 6 previous months (1 patients with rheumatologic inflammatory disease).

For each assessment, the patient’s anthropometric data were collected: birth term, birth height and weight, gender, weight, height, BMI, and Tanner status. For the same patient, all anthropometric measurements were performed by the same operator during follow-up appointments in a standardized manner: for height, with a wall height chart with increments of 0.5 cm; for weight, with a calibrated scale with increments of the nearest 0.1 kg. The Z-scores were calculated for each value according to national data references^21^. Tibia length was measured, the knee flexed at 90%, from the proximal margin of the medial malleolus to the proximal border of the medial tibial condyle (manually located) with a tape measure by the same trained operator. For each patient, IF characteristics and bone complication histories were obtained from medical files: IF etiology and intestinal residual length with or without ileocecal valve, fractures and bone pain.

PN was formulated according to the child’s individual needs.

The study was approved by a local independent ethics committee (*CPP Lyon Sud-Est II, ID-RCB2013-A01245-40*), parental informed consent was obtained for each pediatric patient, and all methods were performed in accordance with the relevant guidelines and regulations**.** The study was registered in Clinical Trial (NCT02368496; 23 February 2015).

### Healthy volunteers

Each patient undergoing PN participating in this study was matched by gender, age and pubertal status (Tanner stage) to two controls recruited concomitantly and locally in the VITADOS cohort of healthy children (NCT 01832623; 16 April 2013), as previously published^22^.

### Biological data

All biological analyses were performed in the same laboratory, immediately after blood sample, in the morning with fasted patients. Usually, in children or adolescent with long-term PN, the frequency of standard biological follow-up is each 3 month when patients are stable. Serum calcium, phosphate, albumin, urinary calcium and creatinine were measured by routine laboratory methods. Intact parathyroid hormone was measured with a second generation assay (Roche Elecsys, Roche Diagnosis, Mannheim, Germany), 25-OH vitamin D with a radio-immunological technique (DiaSorin Assay, DiaSorin Diagnosis, Saluggia, USA), and serum NMID osteocalcin (N-terminal mid fragment of osteocalcin) with immune-chemiluminescence (LIAISON XL, DiaSorin Diagnosis, Saluggia, USA). Glomerular filtration was calculated with the new equation published before^23^.

### HR-pQCT

HR-pQCT measurements were performed at the non-dominant limb unless there was a history of fracture, as previously reported by our team in children^24^. Volumetric bone mineral density (vBMD) and bone microstructure were measured at the distal radius and tibia using a HR-pQCT device (XtremeCT, SCANCO Medical AG, Brüttisellen, Switzerland) that acquires a stack of 110 parallel CT slices with an isotropic voxel size of 82 µm. A scout-view (dorsal–palmar radiography) allowed positioning of a reference line at the endplate. The first slice of the ROI was set at 22.5 mm of the reference line at the ultra-distal tibia and 9.5 mm at the ultra-distal radius, which extended proximally on 110 slices, i.e., 9.02 mm in the axial direction, as previously described. The following imaging settings were used: effective energy = 60 kVp, X-ray tube current = 900µA, integration time = 100 ms. The 126 mm field of view was reconstructed on a 1536 × 1536 matrix, yielding 82 µm isotropic voxels. The total scan time was 2.8 min with an equivalent dose of approximately 3 μSv. Attenuation data were converted to equivalent hydroxyapatite (HA) densities. A phantom was scanned daily for quality control.

For the standard analysis, a trained operator generates semi-automatic contours around the periosteal surface in scans without motion artifact; the entire volume of interest is thereafter automatically separated into a cortical and trabecular region. The outcome variables included total area (Tt.Ar, mm^2^), volumetric bone density (mg HA/cm^3^) for total (Tt.BMD), trabecular (Tb.BMD), and cortical (Ct.BMD) compartments; cortical thickness (Ct.Th, µm); and trabecular number (Tb.N, mm^-1^), thickness (Tb.Th, µm), separation (Tb.Sp, µm), and intra-individual distribution of separation (Tb.Sp.SD, µm). In clinical practice, the higher the trabecular number and thickness, the better the trabecular status; the lower the trabecular separation and distribution, the better the trabecular status.

### DXA: dual X-ray absorptiometry

Whole body DXA and spine DXA were performed with a fan beam (Hologic Discovery W, Hologic, Inc., Bedford, MA) in the array using standard positioning techniques. We performed the standard pediatric protocol of total body less head. The Lumbar spine measurements included L1-L4. The DXA whole body and spine scans were analyzed to generate measures of whole body projected bone area (cm^2^), bone mineral content (BMC) (g) and bone mineral density (BMD) (g/cm^2^). Lean mass (kg) and fat mass (FM) (kg) were obtained from the whole body DXA scan excluding the skull.

### Statistical analysis

Statistical analyses were performed using the SPSS Software. All eligible patients were included. Comparisons between patients and controls were performed using the nonparametric Wilcoxon signed rank test since the number of patients was low. Correlations between density, microarchitectural and biological parameters were computed with the Spearman bivariate analysis. All statistical tests were performed at the two-sided 0.05 level of significance. Data are presented as median, minimum and maximum: med [min–max].

## Results

### Subjects characteristics

We included 11 patients and 22 controls. Anthropometric and clinical data for patients and controls are summarized in Table 1.

**Table 1:** Characteristics of patients and controls.

	Patients	Healthy controls	*p*
**Anthropometry**			
Girls	3	6	
Boys	8	16	
Age (years)	16 [9–19]	16 [10–17]	NS
Weight (kg)	46 [23–73]	56 [31–77]	NS
(SDS)	− 1 [− 3–1]	0.4 [− 1.6–2.5]	< 0.001
Height (cm)	157 [119–173]	168 [131–187]	0.017
(SDS)	− 2 [− 3–0]	0.9 [− 1.5–3]	< 0.001
BMI	19.05 [16.2–25.1]	19.2 [15.5–24.1]	NS
Arm length (cm)	24.5 [18–27]	25.5 [20–31]	NS
Leg length (cm)	34 [26–38]	37 [29–42]	NS
Puberty (tanner)	IV [I–V]	IV [I–V]	NS
**Intestinal characteristics**			
Length of intestine (cm)	90 [30–200]		
Absence of ileocecal valve	1		
**Intestinal insufficiency etiologies n (%)**			
Short bowel syndrome	6 (55%)		
CIPO	3		
Intestinal atresia	3		
Congenital enteropathy	3 (27%)		
Lymphangiectasia	1 (9%)		
Immunodeficiency	1 (9%)		
**Biology**			
Bicarbonate (mmol/L)	27 [22–29]	29 [24–32]	0.003
Albumin (g/L)	38 [25–49]	45 [40–51]	0.015
Pre albumin (g/L)	0.33 [0.13–0.43]	0.26 [0.17–0.32]	0.035
CRP (mg/L)	0 [0–25]	0 [0–2]	NS
Urea (mmol/L)	4 [3–8]	3.5 [2–7]	0.048
Creatinine (µmol/L)	58 [36–98]	64 [37–94]	NS
eGFR (mL/min per 1.73 m^2^)	108 [56–129]	94 [73–129]	NS
Calcemia (mmol/L)	2.29 [2.02–2.5]	2.38 [2.26–2.54]	NS
Phosphoremia (mmol/L)	1.39 [1.02–1.82]	1.29 [1.05–1.79]	NS
Calciuria (mmol/L)	0.94 [0.15–5.26]	2.99 [0.38–12.69]	NS
Ca/creat urinary ratio	0.16 [0.01–1.38]	0.13 [0.02–0.81]	NS
25-OH vitamin D3 (nmol/L)	44 [24–122]	57 [30–123]	NS
Alkaline phosphatase (UI/L)	173 [70–438]	180 [66–311]	NS
Bone alkaline phosphatase (UI/L)	35 [18–98]	71 [13–151]	NS
Serum crosslaps (pmol/L)	1252 [484–2211]	1562 [688–2853]	NS
OC-DiaSorin (µg/L)	44 [15–65]	65 [38–142]	0.005
Parathyroid hormone (ng/L)	41 [7–115]	16 [12–27]	0.003
**Outcome at time of HrpQCT, n (%)**			
Weaning off home TPN	2 (18%)		
Continuing home TPN	9 (82%)		
Death	0		
Intestinal transplantation	0		

Results as median [min–max], CIPO Chronical intestinal pseudo obstruction, Creatinine clearance according to new Schwartz equation (0.413*(height/serum creatinin), CRP: C reactive protein, OC: osteocalcin; SDS: Standard deviation, using national reference growth charts, TPN: Total Parenteral Nutrition.

The median age of initiating PN was 17 (0–150) months, and this nutritional support was given for a median of 10.3 (6.4–18.3) years at the time of this study.

All patients receiving long-term PN had intestinal failure; etiologies are indicated in Table 1.

Two patients at the time of examination had withdrawn PN since 4 months and 5 months respectively. Calories in parenteral nutrition represented a median intake of 77 (40–190) % of recommended dietary allowances. All patients also had oral supplementation: 100 000 UI of vitamin D_3_ each 3 months. Median vitamin D3 intake in PN was 220 (0–250) UI/day. Table 2 summarizes the main PN parameters applied to this cohort.

**Table 2:** Content of parenteral nutrition.

Parenteral nutrition components (median [min–max])	
Age at beginning of PN (months)	17 [0–150]
Mean duration of PN (months)	124 [77–220]
Days/week	5 [3–7]
Days with lipids/week	4 [0–5]
Volume of PN	1500 [1000–3600]
Volume of PN/kg	42 [29–72]
Lipids (g/kg/infusion)	1.5 [0–2]
Carbohydrates (g/kg/infusion)	10 [5–18]
Nitrogen (g/kg/infusion)	0.23 [0.1–0.4]
Calcium (mmol/kg/infusion)	0.35 [0–0.88]
Magnesium (mmol/kg/infusion)	0.3 [0.1–0.6]
Phosphorus (mmol/kg/infusion)	0.6 [0.3–1]
Acetates (mmol/kg/infusion)	1 [0–2]
CERNEVIT (bottle/perfusion)	1 [0–2.5]
Vitamin D3 (UI/perfusion)	220 [0–550]
Trace elements (mL/kg/perfusion)	2 [0–2]
Calories (kcal/kg/infusion)	53.5 [25–90]
%PN RDA	77 [41–190]

Results as median [min–max], PN: parenteral nutrition, RDA: recommended dietary allowances.

Three patients had also enteral nutrition during PN or during nights without PN.

Four patients and one control had history of long bone fracture, none had a vertebral fracture and none had more than two fractures. All were traumatic fractures.

### Biological parameters

Biological parameters for patients and controls are also summarized in Table 1. Nutritional biological parameters (albumin, prealbumin, urea, creatinin and eGFR according to the recent equation) were normal in the 2 groups; however compared to controls, PN patients displayed lower median albumin levels (84% of controls; *p* = 0.015) but higher prealbumin (127% of controls; *p* = 0.035) and urea levels (114% of controls; *p* = 0.048). Parameters of calcium/phosphate and bone metabolism (calcium, phosphate, urinary calcium to creatinine ratio, 25 hydroxy-vitamin D and total alkaline phosphatase) were similar between the two groups. However, PN patients displayed higher but normal PTH (256% of controls; *p* = 0.003) and lower but normal osteocalcin plasmatic levels (67% of controls; *p* = 0.005), as compared to controls.

### HR-pQCT

At the radius, no significant differences were observed between controls and PN patients, as illustrated in Table 3. However at the tibia, PN children displayed a lower trabecular area compared to controls (470 [124–675] vs 606.5 [369–897] µm^2^, *p* = 0.003).

**Table 3:** HR-pQCT and DXA values between patients and healthy controls.

HR-pQCT	RADIUS			TIBIA		
	Patients	Healthy controls	*p*	Patients	Healthy controls	*p*
**Cortical**						
Ct-Th (µm)	505 [50–980]	480 [130–1080]	NS	1100 [280–1390]	1010 [280–1480]	NS
Ct-Ar (µm^2^)	33 [3–67]	28 [8–81]	NS	82 [29–134.4]	108 [25–169]	NS
Ct-vBMD (mg/µm^3^)	696 [450–859]	699 [524–887]	NS	815 [641–898]	768 [606–912]	NS
**Trabecular**						
Tb-Ar (µm^2^)	196 [110–288]	244 [120–360]	NS	470 [124–675]	607 [369–897]	**0.003**
Tb-vBMD (mg/µm^3^)	215 [166–333]	199 [122–298]	NS	316 [176–389]	283 [218–368]	NS
Tb-Th (µm)	790 [730–1160]	830 [620–990]	NS	77 [71–108]	87 [77–106]	NS
Tb.Sp (µm)	370 [301–456]	418 [296–546]	NS	482 [324–670]	447 [326–579]	**0.063**
**Total**						
Total-vBMD (mg/µm^3^)	303 [223–415]	276 [187–376]	NS	316 [176–389]	283 [218–368]	NS

DXA	Patients	Healthy controls	*p*			
**SPINE total**						
Area (cm^2^)	51 [33–70]	61 [38–71]	0.048			
BMC (g)	40 [14–66]	56 [20–97]	NS			
BMD (g/cm^2^)	0.8 [0.4–0.9]	0.9 [0.5–1.4]	NS			
**Whole body**						
Area (cm^2^)	1480 [820–1986]	1795 [1077–2319]	NS			
BMC (g)	1198 [441–1832]	1638 [713–3129]	NS			
BMD (g/cm^2^)	0.81 [0.54–0.96]	0.92 [0.66–1.35]	0.039			

Results as median [min–max], Ar: Area, Ct: cortical, BMC: bone mineral content, BMD: bone mineral density, Ct-Ar: cortical area, HR-pQCT: high resolution peripheral quantitative computed tomography, Tb: trabecular, TbN: trabecular number, TbSp: trabecular separation, TbSpSD: heterogeneity of trabecular separation, TbTh: trabecular thickness, Th: thickness, vBMD: volumetric bone mineral density, *p* < 0.05 significant. Of note: tota body scan excluded the head.

### DXA and body composition

Body composition data are summarized in Tables 3 and 4. BMC was similar to controls; only the BMD of the whole body was significantly lower in patients (0.81 [0.54–0.96] vs 0.92 [0.66–1.35], *p* = 0.039). In terms of body composition, the global distribution was similar between patients and healthy patients. However patients differed according the gynoid lean and total mass distribution. The android-gynoid ratios and the BMI were similar to the controls.

**Table 4:** Comparisons of data measurements of body composition by DXA between patients and healthy controls.

	Patients	Healthy controls	*p*
Subtotal fat (g)	8048.87 [4881.2–15,698.5]	9106.7 [5591.6–18,676.3]	NS
Subtotal lean (g)	37,992.2 [15254.6–58,414.1]	48,237.1 [24213.4–70,779.9]	NS
Subtotal mass (g)	45,188.6 [23457.1–74,112.6]	56,505.3 [31797.6–78,710.9]	NS
Android fat (g)	453.6 [234.9–892.6]	456.9 [215.9–1096.2]	NS
Android lean (g)	2664.7 [1052.8–4586.9]	2959.8 [1440.1–4553.9]	NS
Android mass (g)	3214.6 [1531.6–5479.5]	3473.3 [1796.5–4999.9]	NS
Gynoid fat (g)	1356.3 [734.1–3029.3]	1734.5 [921.2–3885.3]	NS
Gynoid lean (g)	5286.7 [1843.8–8338.4]	7216.1 [3016.9–10,935.1]	0.047
Gynoid mass (g)	6509.4 [3238.4–11,367.7]	8881.8 [4443.9–12,669.6]	0.025
Android-gynoid ratio	0.67	0.74	NS

Results as median [min–max], BMC: bone mineral content, BMD: bone mineral density.

## Discussion

This pilot prospective study is the first to evaluate bone microarchitecture with HR-pQCT in pediatric patients undergoing long-term PN in comparison to healthy controls. The main results are: (1) reassuring results for bone quality and body composition evaluated by both HR-pQCT (with similar densities and microarchitecture between PN patients and controls) and DXA (with BMC similar to controls); and (2) higher but normal PTH levels despite correct vitamin D levels, with lower but normal osteocalcin levels in PN patients as compared to controls.

### PN-associated metabolic bone disease

The main previous studies that have described bone diseases in patients receiving home PN were adult studies^5,8,11^. The first pediatric study was published in 2010^25^. Of note, 83% of these patients had bone mineral deficiency and 17% had fractures. Different studies evaluated longitudinal evolution and predictive factors, including intestinal failure-related factors and parenteral nutrition-related factors^15,25–27^. Risk factors identified were: (1) the etiology of intestinal failure: patients with a small bowel syndrome were more at risk for bone disease; (2) the duration of PN was correlated to lower lumbar BMD; (3) others factors such as the small bowel length and the presence of the ileocecal valve did not predict BMD.

Compared to previous pediatric studies, our cohort had less PN-MBD (only 2 (18%) patients had a whole body BMC ≤ − 2 DS). Several reasons may explain this discrepancy: the etiologies are different since we excluded patients with inflammatory bowel disease and patients having received steroid therapy for more than 1 month during the 6 previous months. These etiologies differ from other studies describing lower bone mineral density in patients on long-term PN which may include inflammatory bowel disease^4,8,9,11^. We can also hypothesize that the treatment and patient care were effective in preventing bone deterioration.

A significant fracture history is defined by either one or more vertebral crush fractures, two or more long bone fractures by 10 years of age, or three or more fractures by 19 years of age^12^. The clinical bone history of our patients indicates that four patients had a past of bone fracture, and only one control subject. However, as noted below, radiological exams (HR-pQCT and DXA) were not significantly different between the two groups. It seems unlikely that all radiological measurements are ineffective to identify the risk of bone fracture, and we face here one of the main limitations of this cohort namely its limited size.

Determination of the density and bone microarchitecture in children and teenagers with long-term PN.

Compared to DXA, the main objective of HR-pQCT is to evaluate microarchitectural composition. Low trabecular area and high trabecular separation are associated with an increased risk of osteoporosis. This profile associated with bone fragility is described only at the ultra-distal tibia of our patients, and only with a small difference. However, there are no significant differences in cortical parameters that are involved more in bone strength than trabecular ones. Many studies have shown an increased cortical vBMD with age without modifications of trabecular parameter^18,28,29^. Cortical vBMD measured by HR-pQCT increased with puberty in both genders^28^. Therefore, this small difference reported only at the ultra-distal tibia of patients should have only a minor effect if any on bone solidity.

### Comparison of HR-pQCT results to DXA measurements

Our study seems to indicate very mild differences of mineral status with both radiological techniques when compared patient to healthy controls. On one hand, HR-pQCT indicates no difference in BMD but only minor trabecular changes at the tibia; on the other hand DXA indicates no alteration of the main parameters (namely spine BMD, BMC or whole body BMC that correspond to the recommended measure sites in pediatrics)^30^.

BMD had divergent results with the two radiological techniques: HR-pQCT measured similar total volumetric BMD in patients and controls. In contrast, DXA showed a significant lower whole body subtotal BMD in patients. This may suggest that (1) inherent differences between the radiological techniques (HR-pQCT versus DEXA); (2) BMD of the ultra-distal radius and tibia do not reflect whole body BMD, but previous studies have found DXA spine and hip scans do correlate with HR-pQCT radius and tibia scans; (3) HR-pQCT and DXA evaluate bone composition differently; (4) our patients were smaller than controls but had a similar BMI. That could explain a lower BMD with DXA. DXA relies on areal density rather than volumetric density that can be modified by growth. Therefore, the risk of underestimating BMD in small children and overestimating BMD in tall children is well known^13,31^.

### Evaluation of the biological markers of bone metabolism

Very few pediatric studies accurately assessed the biological markers of bone metabolism in PN. We did not find biological abnormalities in this study for the markers of bone metabolism; however, as compared to controls, our patients displayed different levels of PTH and osteocalcin that however both remained within the normal range.

Indeed, patients displayed a relative hyperparathyroidism: PTH levels were within the normal range, but significantly higher in patients compared to controls. Hyperparathyroidism in patients on long-term PN has been previously described^26,32^. A relationship between hyperparathyroidism and bone remodeling was also previously described in adults^33,34^. In our patients, this higher level of PTH is not explained by a lower vitamin D level, which is similar to healthy controls. Based on international recommendations, a partial deficiency could be considered in patients and controls since vitamin D levels should be higher than 75 nmol/L^35^. The recommendation for parenteral vitamin D intakes are 400 UI/day^36^. Our patients received only a median intake of 268 ± 0.87 UI/day in perfusion, but also an oral supplementation of vitamin D every 3 months. Therefore, intestinal malabsorption could explain their low plasmatic level of vitamin D; this result should encourage increasing oral doses and closely monitoring plasmatic vitamin D.

Low calcemia and high phosphoremia could explain a higher PTH. This hormone increases calcium levels and decreases phosphate levels, through a stimulation of 1–25 OH2 vitamin D synthesis and further activation of intestinal calcium absorption, through a stimulation of tubular calcium reabsorption and through an inhibition of the apical expression of the sodium/phosphate transporters Npt2 thus inducing phosphaturia. Last, PTH also has biphasic effects on bone, depending on the levels of PTH and its pulsatility. In our patients, calcium and phosphate parenteral intakes are within the range of the recommendations Calcium 0.35 for 0.25–0.4 mmol/kg/day and phosphate 0.6 for 0.2 to 0.7 mmol/kg/day, whereas the rates of calcemia and phosphatemia were similar in the two groups^37^. Unfortunately, we do not have urinary calcium and phosphate levels for all patients to evaluate tubular reabsorption. However, the Ca/creat urinary ratio was normal and not different between patients and controls.

Finally, an acid–base imbalance could also increase PTH levels. Indeed, metabolic acidosis rapidly inhibits the CaSR that causes PTH release and relative hyperparathyroidism. Acid–base balance was not specifically studied in this cohort, even if PN patients displayed significant lower plasma bicarbonate concentrations in comparison to controls. However, the values are normal and the difference does not seem clinically relevant.

### Osteocalcin

Patients had a significantly lower osteocalcin levels than controls. Osteocalcin is produced by differentiated osteoblasts. Furthermore, non-carboxylated osteocalcin regulates glucose homeostasis by increasing insulin secretion and decreasing insulin resistance.

Osteocalcin level is influenced by different parameters: (1) gender: higher in women; (2) age with a decrease with age in both men and women, and especially during puberty^38^; (3) physical activity: higher after a run. As our patients are gender- and age-matched with controls, variations of osteocalcin could indicate an impaired glucose metabolism^38–40^. Unfortunately, we do not have the parameters to evaluate this hypothesis such as, for example, the Homeostasis Model Assessment (HOMA) scale of insulin resistance (insulin (mUI/L) × glucose (mmol/L)/22.5)^41–43^. It would be interesting for a future study to collect insulin and glucose levels in these patients.

Several limitations of this study may be highlighted. It is a monocentric study with a small cohort. However, this study was designed as a pilot study, and children receiving long-term PN are rare. Another limitation in the interpretation of results is the significant difference observed in height and body weight between patients and controls, likely explaining the lower trabecular area observed at the weight-bearing tibia in PN patients. Body size differences alone could account for any discrepancies between the clinical and healthy groups, an adjustment for height between patients and controls would ideally be useful. Even though we selected the smallest subjects among the controls (after matching for gender, age and puberty), it was impossible to adjust for height given the difference of height between controls and patients. However, odd differences in height between groups is little, and limb length is similar between the 2 groups; therefore we believe that these two limitations are not crucial in view of the observed results, namely the absence of significant differences in bone status between controls and PN patients^28,44,45^.

## Conclusion and perspectives

This first study evaluating microarchitecture in children receiving long-term PN provides quite reassuring results: the innovative imaging technique HR-pQCT did not find any deterioration of bone outcomes in our cohort after more than 10 years of PN. However, the small sample size requires caution in interpretation. Further longitudinal studies, with larger cohorts, are required to confirm these data and to determine if such techniques could help physicians improve the therapeutic management of children receiving long-term PN.
